# Regulatory Effects of Astragaloside IV on Hyperglycemia-Induced Mitophagy in Schwann Cells

**DOI:** 10.1155/2022/7864308

**Published:** 2022-01-11

**Authors:** Xiaoyi Wei, Yalin Zheng, Yanke Ai, Buman Li

**Affiliations:** ^1^Beijing Key Lab of TCM Collateral Disease Theory Research, School of Traditional Chinese Medicine, Capital Medical University, Beijing 100069, China; ^2^Institute of Basic Research in Clinical Medicine, China Academy of Chinese Medical Sciences, Beijing 100700, China; ^3^Tsinghua University Yuquan Hospital, Beijing 100040, China

## Abstract

**Objective:**

This study aimed to observe the regulatory effects of astragaloside IV (AS-IV) on hyperglycemia-induced mitochondrial damage and mitophagy in Schwann cells and to provide references for clinical trials on AS-IV in the treatment of diabetic peripheral neuropathy.

**Methods:**

Schwann cells were grown in a high-glucose medium to construct an autophagy model; the cells were then treated with AS-IV and N-acetylcysteine (control) to observe the regulatory effects of AS-IV on oxidative stress and mitophagy.

**Results:**

AS-IV exhibited antioxidant activity and inhibited the overactivation of autophagy in Schwann cells, significantly reducing the level of reactive oxygen species and downregulating the expression of autophagy-related proteins (LC3, PINK, and Parkin) under hyperglycemic conditions, thereby exerting a protective effect on mitochondrial morphology and membrane potential.

**Conclusion:**

AS-IV can maintain the mitochondrial function of Schwann cells under hyperglycemic conditions by effectively alleviating oxidative stress and overactivation of mitophagy. The evidence from this study supports an AS-IV-based therapeutic strategy against diabetic peripheral neuropathy.

## 1. Introduction

Diabetic peripheral neuropathy (DPN) is a chronic diabetes complication. Due to its high incidence and severe disability rate, the prevention and treatment of DPN have become a crucial topic in diabetes research. Previous research has demonstrated that the pathogenesis of DPN primarily includes metabolic alterations, changes in immune functions, ischemia, inflammation, and oxidative stress, among which oxidative stress has been widely considered to play a vital role in the pathogenesis of DPN [1, 2]. Therefore, antioxidant therapies targeting oxidative stress may be critical for DPN treatment.

Nerve tissue is vulnerable to oxidative stress damage as it is rich in mitochondria. During oxidative stress, cells must undergo selective autophagy, called mitophagy, to remove damaged organelles, such as mitochondria, whereby intracellular stress is regulated by reducing the levels of reactive oxygen species (ROS) produced by mitochondria to protect cells from ROS-mediated damage. Autophagy is essential in maintaining the normal morphological structure and physiological functions of neurons. The dynamic equilibrium between the formation and degradation of autophagosomes is maintained during autophagy under normal conditions. By contrast, the equilibrium shifts toward autophagosome formation under pathological conditions, such as cell injuries and starvation, leading to autophagosome buildup, called autophagic stress [3, 4]. Neurons are highly sensitive to increased autophagic stress, whereby an abrupt elevation can cause drastic intracellular changes that further enhance autophagic activity in neurons. This results in necrosis and neuron disintegration, as well as various neurological diseases, such as Alzheimer's, Parkinson's, and peripheral neurodegenerative diseases caused by Schwann cell (SC) autophagy [5, 6].

SCs are a critical component of the peripheral nervous system and play a role in axonal growth, regeneration, and myelination [7]. They also secrete various neurotrophic factors associated with nerve regeneration, such as the brain-derived neurotrophic factor, nerve growth factor (NGF), neurotrophic factor-3, and glial cell-derived neurotrophic factor [8, 9], which can promote the survival of injured neurons. In vitro and in vivo studies demonstrated that DPN is closely associated with hyperglycemia, and long-term hyperglycemic exposure can lead to peripheral nerve demyelination and SC apoptosis [10], implicating the protection of SCs as a potential strategy for the treatment of DPN.

Astragaloside IV (AS-IV) is a natural saponin isolated from *Astragalus membranaceus*. Studies have suggested its antioxidant, anti-inflammatory, and antiapoptotic activities as well as its regulatory effects on autophagy [11]. AS-IV also exerts a protective effect against damage to the liver, kidneys, retina, and other tissues of diabetic rats [12] and an antiapoptotic effect on dorsal root ganglion cells in rats with DPN [13]. However, whether AS-IV has a direct protective effect on SCs remains unclear. Therefore, we examined the effects of AS-IV on autophagic signaling pathways and the morphology and functions of the mitochondria in SCs to investigate its protective effect on SCs and molecular mechanism under hyperglycemic conditions. Thus, we provide a reference for studying the therapeutic value of AS-IV against diabetic peripheral nerve injuries.

## 2. Materials and Methods

### 2.1. Cultivation of RSC96 Cells

Cells of the rat Schwann cell line RSC96 (BNCC341121; Beijing Pujing Kangli Technology Co., Ltd.) were inoculated into 7 mL of Dulbecco's modified Eagle medium (DMEM, containing 4.5 g/L D-glucose, L-glutamine, and 110 mg/L sodium pyruvate; Gibco) supplemented with 10% fetal bovine serum (Gibco, 10270–106) and 5% penicillin-streptomycin (Keygentec, KGY002) in T25 culture flasks. Next, the flasks were shaken vigorously to evenly disperse the cells and then incubated at 37°C with 5% CO_2_. The cell culture was then passaged after reaching 70% confluence when observed under the microscope.

### 2.2. Determination of the Dosage of Hyperglycemic Exposure for the Construction of the Hyperglycemia-Induced Autophagy Model

**Figure 1:**
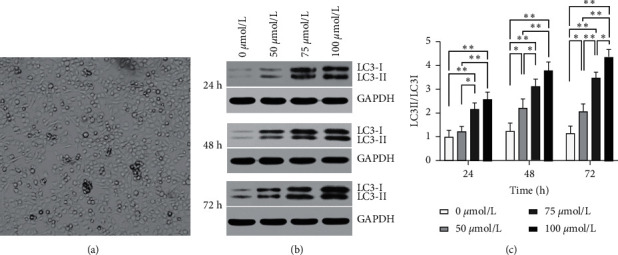
Determination of the dose and duration of hyperglycemic exposure for constructing the hyperglycemia-induced autophagy model. (a) Two-day-old culture of Schwann cells. (b) Determination of the expression levels of LC3II and LC3I proteins 24, 48, and 72 h postexposure via Western blot assays. (c) Expression level of LC3 at different concentrations and duration of exposure to glucose. Statistical significance: ^*∗*^*p* < 0.05, ^*∗∗*^*p* < 0.01.

First, the cells were exposed to different glucose concentrations (50, 75, and 100 mmol/L). They were then harvested 24, 48, and 72 h postexposure to determine the dosage of hyperglycemic exposure for construction of the hyperglycemia-induced autophagy model. The model was constructed by comparing the expression level of LC3 proteins via Western blot (WB) assay on LC3II/LC3I. The results of WB assays showed that exposure to 100 mmol/L of glucose for 72 h was optimal for constructing the autophagy model (Figure 1).

### 2.3. Treatment of Cells in Different Groups

The cells were divided into four groups: normal control (NC), wherein the cells were treated with an equal volume of DMEM complete medium; hyperglycemic (HG), wherein the cells were treated with an equal volume of DMEM high-glucose complete medium (containing 100 mmol/L of glucose); AS-IV, wherein the cell model of hyperglycemia was treated with 50 *μ*mol/L of AS-IV (H-013; Chengdu Herbpurify Co., Ltd.); and N-acetylcysteine (NAC), wherein the cell model of hyperglycemia were treated with 100 *μ*mol/L of N-acetylcysteine (G-Clone Biotechnology, Tianjin Co., Ltd.). The cells in each group were then harvested after 72 h of treatment.

### 2.4. Flow Cytometric Determination of ROS

After discarding the culture supernatant, the cells in each treatment group were treated with dichlorodihydrofluorescein diacetate (1 : 1000 dilution with serum-free medium; S0033; Beyotime Institute of Biotechnology Co., Ltd.), incubated at 37°C for 20 min, and rinsed with a serum-free medium. A flow cytometric measurement of ROS was conducted using the BD FACSCalibur flow cytometer with excitation and emission wavelengths of 488 nm and 525 nm, respectively.

### 2.5. Transmission Electron Microscopic Analyze of Mitochondria

The cells were collected and fixed in ice-cold glutaraldehyde (2.5% in 0.1 mol/L cacodylate buffer, pH 7.4) for 24 h. The following day, the cells were postfixed with 1% osmium tetroxide for 2 h. After dehydration with a graded series of alcohol concentrations, the samples were rinsed in propylene oxide and impregnated with epoxy resins, and frontal sections were cut. The cells were then stained with 2% uranyl acetate in 50% ethanol and lead citrate for microscopic evaluation. The ultrastructure was examined using a transmission electron microscope (JEM1230; JEOL Ltd., Japan).

### 2.6. Flow Cytometric Determination of Mitochondrial Membrane Potential (MtMP)

A total of 6 × 105 cells harvested from each treatment group were resuspended in 0.5 mL of medium, mixed thoroughly with 0.5 mL of JC-1 staining solution (C2006; Beyotime Institute of Biotechnology Co., Ltd.), and incubated at 37°C for 20 min. JC-1 staining solution (5X) was diluted with distilled water at a ratio of 1 : 4 to prepare an appropriate amount of 1X JC-1 staining solution, which was then placed in an ice bath until use. After incubation, the cell suspension was centrifuged at 600 × g and 4°C for 3-4 min, and the resulting supernatant was discarded. The cell pellet was rinsed twice with 1X JC-1 staining solution and resuspended with an appropriate amount of the staining solution. The flow cytometric measurement of MtMP was conducted using the BD FACSCalibur flow cytometer.

### 2.7. Observation of Autophagic Flux via Laser Confocal Scanning Microscopy (LCSM)

The cells harvested from each treatment group were rinsed with phosphate-buffered saline (PBS) supplemented with 5% FBS, mixed with the Cyto-ID staining solution (ENZ-KIT175-0200; Enzo Life Sciences, Inc., USA), and incubated in the dark at 37°C for 30 min. The cells were then subjected to nuclear staining with Hoechst 33342 blue fluorescent dye (Sigma-Aldrich Pty. Ltd.) and rinsed twice with PBS (FBS-free) to remove any dye residue. Finally, the cells were observed and imaged using a Zeiss LSM800 laser confocal scanning microscope.

### 2.8. Immunoblotting Analysis of LC3 and Parkin

The cells were washed with PBS and lysed in radioimmunoprecipitation assay buffer and protease inhibitor (Applygen Technologies, China) according to the manufacturer's instructions. All steps of protein extraction were performed on ice. The lysates were assayed using the bicinchoninic acid kit to determine the protein concentration. Equal amount of protein samples (20–80 *μ*g) were separated using 10–12% gradient polyacrylamide gels (Bio-Rad) and then electrotransferred to polyvinylidene fluoride membranes. After blocking with 5% skim milk for 1 h, the membranes were incubated with the specific antibodies (LC3: ab48394, Abcam, USA; Parkin: ab77924, Abcam, USA; PINK: ab23707, Abcam, USA; GAPDH: ab181602, Abcam, USA) overnight at 4°C. Following three washes with Tris-buffered saline with Tween-20 (Applygen Technologies, China), the membranes were incubated with a secondary antibody for 1 h at room temperature. All primary antibodies were diluted at 1 : 1000, and the corresponding horseradish peroxidase-conjugated secondary antibodies were diluted at 1 : 5000. After three washes with TBST, the blot was visualized and subjected to quantification analysis using Quantity-One (Bio-Rad, the USA) software.

### 2.9. Statistical Analysis

All data are presented as the mean ± standard deviation (SD). One-way analysis of variance was performed to compare the variables among multiple groups. Statistical tests were performed using the GraphPad Prism 8.0 software, and statistical significance was set at *p* < 0.05.

## 3. Results and Discussion

### 3.1. Determination of the Dosage of Hyperglycemic Exposure for the Construction of the Hyperglycemia-Induced Autophagy Model

In this study, we first treated cells with 0, 50, 75, and 100 mmol/L of glucose for 24, 48, and 72 h to determine the optimal concentration and duration of exposure for constructing the autophagy model. Next, we compared the autophagy level between groups based on LC3II/LC3I expression levels determined via WB assays and found that exposure to 100 mmol/L of glucose for 72 h is optimal for constructing the autophagy model (Figure 1).

### 3.2. Evaluation of Antioxidant Activity Using ROS Assay

**Figure 2:**
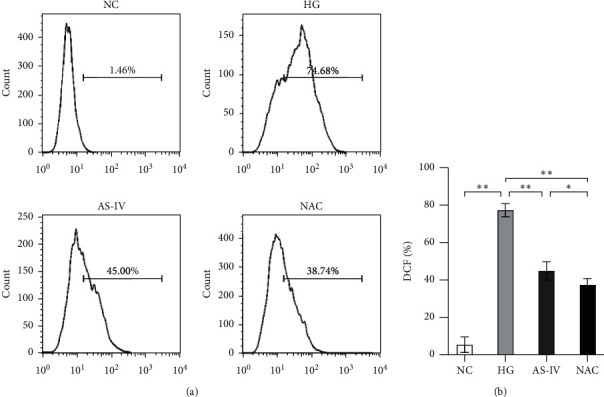
Flow cytometric determination of the intracellular reactive oxygen species (ROS) level in each group. (a) ROS level of each group. (b) Statistical results of ROS level (^*∗*^*p* < 0.05, ^*∗∗*^*p* < 0.01).

As shown in Figure 2, SCs displayed significantly increased ROS levels under hyperglycemic conditions, and significant differences were observed between the HG and NC groups (*p* < 0.01). ROS levels in the AS-IV and NAC groups were significantly lower than those in the HG group (*p* < 0.01). In addition, the NAC group had a significantly lower ROS level than the AS-IV group (*p* < 0.05). The results indicate that AS-IV exhibits antioxidant activity and significantly reduces the ROS level in SCs under hyperglycemic conditions.

### 3.3. Electron Microscopic Observation of Mitochondrial Morphological Changes in Different Groups

**Figure 3:**
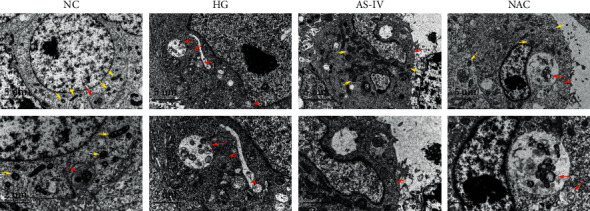
Electron microscopic observation of the morphological changes in mitochondria. The yellow arrows indicate morphologically normal mitochondria, whereas the red arrows indicate morphologically abnormal mitochondria. Scale bar: 5 *μ*m, 2 *μ*m.

The SCs in the NC group had morphologically intact and distinct mitochondria, whereas those in the HG group showed swollen, deformed, and fragmented mitochondria with abundant vacuoles and double-membrane autophagic vesicles in the cytoplasm. Some mitochondria were deformed, and a small number of autophagic vesicles were observed in SCs of the AS-IV and NAC groups (Figure 3). These results suggest that AS-IV has a protective effect on the mitochondrial morphology of SCs under hyperglycemic conditions.

### 3.4. Effect of AS-IV on MtMP

**Figure 4:**
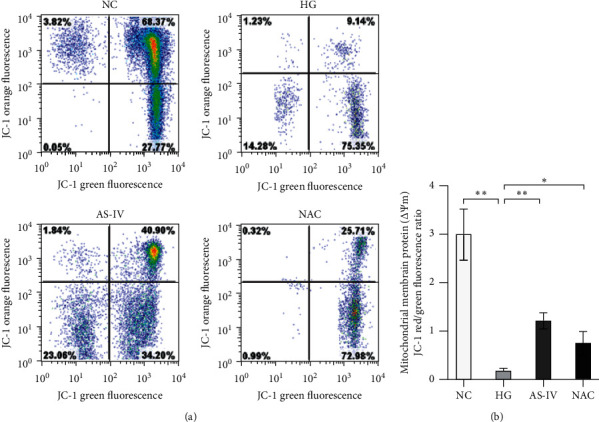
(a) Flow cytometric measurement of mitochondrial membrane potential. (b) The results of MtMP. Data are presented as the mean ± SD of three independent experiments. Statistical significance: ^*∗*^*p* < 0.05; ^*∗∗*^*p* < 0.01.

Mitochondrial damage manifested as a decrease in MtMP, resulting in the alteration of mitochondrial oxygen consumption rate (OCR) and decreased ATP production. Therefore, the MtMP of SCs was detected to examine the effect of AS-IV on mitochondrial damage under a high glucose environment. At higher MtMP concentrations, JC-1 accumulated in the mitochondrial matrix to form a polymer that emitted orange fluorescence. At lower concentrations, JC-1 was unable to accumulate in the mitochondrial matrix and remained as a monomer that emitted green fluorescence. The MtMP of SCs of four groups was detected by flow cytometry. The degree of mitochondrial depolarization in each group was measured based on the ratio of the orange-to-green fluorescence intensity (Figure 4(a)). The results showed that MtMP of SCs in the HG group was significantly lower than that in the NC group (*p* < 0.01). MtMP increased after treatment with AS-IV and NAC, and SCs in the AS-IV group displayed significantly higher MtMP than those in the HG group (*p* < 0.01; Figure 4(b)). The results indicate that AS-IV has a protective effect on mitochondrial functions and effectively improves the MtMP of SCs exposed to a high glucose environment.

### 3.5. Observation of Autophagic Flux under LCSM

**Figure 5:**
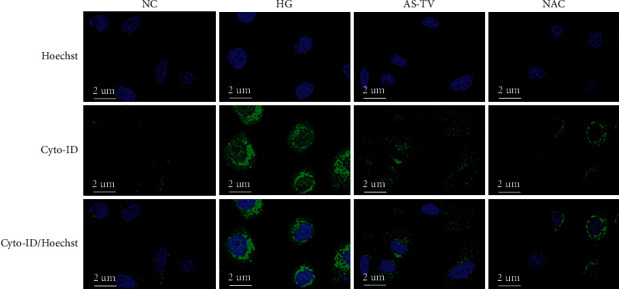
Observation of autophagic flux under laser confocal scanning microscopy. Scale bar: 2 *μ*m.

Hoechst stain is a blue fluorescent dye for nuclear staining, and Cyto-ID dye selectively labels accumulated autophagic vesicles and emits bright green fluorescence in autophagosome precursors, autophagosomes, and autophagolysosomes. As shown in Figure 5, the HG group displayed higher green fluorescence intensity and density than the NC group, suggesting the presence of abundant autophagic vesicles and enhanced autophagic flux in SCs of the HG (model) group. By contrast, the AS-IV and NAC groups showed reduced green fluorescence intensity and density. These results suggest that AS-IV can significantly inhibit the overactivation of autophagy in SCs exposed to a high glucose environment.

### 3.6. Determination of the Expression Levels of Autophagy-Related Factors by Western Blot

**Figure 6:**
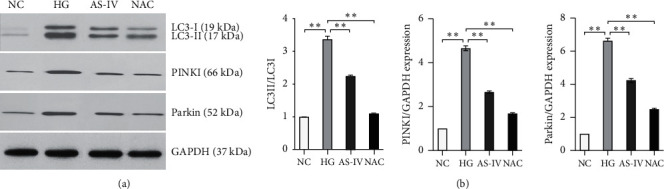
Western blot assays on the expression levels of LC3, PINK, and Parkin (^*∗*^*p* < 0.05, ^*∗∗*^*p* < 0.01).

Expression levels of the autophagy-related proteins LC3, PINK, and Parkin were analyzed by Western blot (Figure 6(a)). These protein expressions were significantly increased in SCs of the HG group (*p* < 0.01) but declined after intervention with AS-IV and NAC, with the AS-IV group showing significantly lower protein expression levels than the HG group (*p* < 0.01) and significantly higher protein expression levels than the NAC group (*p* < 0.01) (Figure 6(b)). The results indicate that AS-IV can downregulate the expression of autophagy-related proteins, such as LC3, PINK, and Parkin, in SCs grown in a high-glucose medium.

DPN is correlated with hyperglycemia, which affects its onset and development in several ways. Studies have demonstrated that high glucose concentrations adversely affect apoptosis, autophagy, metabolism, proliferation, migration, and NGF secretion. High glucose levels can induce apoptosis in SCs [14, 15] in a concentration-dependent manner [16]. Rats with DPN have a reduced surface area of myelin [17] and reduced levels of autophagy markers as a result of the loss and morphological abnormality of myelinated nerve fibers [18]. In addition, hyperglycemia can reduce the viability and inhibit the proliferation of SCs [19, 20]. Furthermore, increased inflammatory response may lead to nerve damage in diabetes. Suppressing TGF-*β* upregulation and targeting the RAP1/KC/NLRP3 inflammasome may be potential therapeutic strategies for DPN [21, 22]. These suggest that the protective effect of AS-IV on DPN may occur via multiple mechanisms. Our in vitro study reveals that hyperglycemic exposure may lead to autophagy and ROS production in RSC96 cells, which is consistent with the findings of the study by Liu et al. [23].

Autophagy is a tightly regulated cellular mechanism with beneficial and pathogenic outcomes [24, 25]. It has a protective effect that can create a favorable environment for cell survival under physiological conditions, inhibiting cell apoptosis by eliminating damaged organelles or subcellular components. For example, autophagy levels in SCs increased significantly after sciatic nerve injury [26]. However, overactivation of autophagy has been reported to potentially promote oxidative stress [27, 28] and induce apoptosis [29]. Oxidative stress is an important factor that leads to injuries of SCs [30]. ROS are highly reactive metabolites produced during the normal cellular metabolism [31]. However, ROS accumulation promotes autophagy and apoptosis [32, 33], and increased levels have been shown to lead to mitochondrial dysfunction and axonal degeneration [34], damaging the lipids in myelin and the microvascular system in peripheral nerves. SCs are the main glial cells of the peripheral nervous system. Some studies have posited that the scavenging rate of damaged myelin can be improved by regulating autophagy in SCs [35]; however, the effect on subsequent axon regeneration remains controversial [36]. We hypothesize that the key to this phenomenon is the modulation of the intracellular environment to favor survival by balancing the degree of autophagy (i.e., the equilibrium between inactivation and overactivation) during SC damage caused by hyperglycemia. Mitophagy is primarily induced by PINK proteins, constantly degraded by presenilin-associated rhomboid-like protein (PARL) under healthy conditions. The stabilized PINK proteins recruit the E3 ligase Parkin to induce autophagy after the inhibition of the PARL protein in the event of mitochondrial damage.

Our study revealed that hyperglycemia induced oxidative stress and continuous production of ROS, significantly upregulating the expression of autophagy-related proteins (LC3, PINK, and Parkin) in SCs. However, autophagy did not alleviate hyperglycemia-induced cellular damage. Instead, the overactivation of autophagy resulted in varying degrees of morphological and functional damage to the mitochondria in SCs. The intracellular level of ROS and the expression levels of autophagy-related proteins declined along with morphological and functional improvements in mitochondria after intervention with the antioxidant drug NAC. The results indicated that the autophagy level was positively correlated with the degree of oxidative stress in SCs continuously stimulated with high glucose, during which the cells were exposed to both oxidative stress and excessive autophagy. Hence, antioxidation and inhibition of autophagy overactivation are equally crucial in protecting SCs.

AS-IV is the main active ingredient of *Astragalus*, which is used in traditional Chinese medicine and is associated with biological activities, such as antiapoptosis, anti-inflammation, and immunomodulation. In addition, *Astragalus* has exhibited a protective effect against nerve injuries [37, 38] and damage to other tissues, such as the liver, kidneys, and retina, in diabetic rats [12]. However, whether AS-IV has a direct protective effect on SCs remains unclear. NAC can be used as a common antioxidant agent due to its free radical scavenging activity [39]. Therefore, we used NAC as a control to observe the therapeutic efficacy of AS-IV. The results show that AS-IV has a regulatory effect on the oxidative stress in SCs under hyperglycemic conditions and can alleviate the overactivation of mitophagy caused by the increased ROS levels, exerting a protective effect on SC mitochondrial function. Therefore, our study provides a valuable reference for investigating the therapeutic value of AS-IV in the treatment of DPN.

Despite its value, our study has some limitations. Because of temporal and financial constraints, we conducted only in vitro assays on RSC96 cells, not in vivo assays using rat models. Therefore, the therapeutic effect of AS-IV against DPN and its regulatory effect on autophagy in SCs need to be confirmed using in vivo assays in future studies. In addition, OCR production and ATP production are important parameters for evaluating mitochondrial function [40, 41]. Experiments reflecting the mitochondrial function of SCs can be further improved, such as by testing the abovementioned indicators to evaluate mitochondrial function after treatment.

## 4. Conclusions

This study explored the regulatory effect of AS-IV on hyperglycemia-induced mitochondrial damage and mitophagy in SCs and found that AS-IV maintains the mitochondrial function of SCs under hyperglycemic conditions by alleviating oxidative stress and overactivation of mitophagy. However, our study also has some limitations. In the future, in vivo experiments on a DPN model should be conducted to further investigate the mechanism of AS-IV treatment for DPN.

## Data Availability

The datasets used or analyzed in the current study are available from the corresponding author upon request.
